# Clinical and molecular epidemiology of carbapenem-resistant Enterobacteriaceae in pediatric inpatients in South China

**DOI:** 10.1128/spectrum.02839-23

**Published:** 2023-10-11

**Authors:** Zhile Xiong, Chao Zhang, Kurosh Sarbandi, Zhuwei Liang, Jialiang Mai, Bingshao Liang, Hao Cai, Xiantang Chen, Fei Gao, Fangjun Lan, Xiaochun Liu, Shuyan Liu, Zhenwen Zhou

**Affiliations:** 1 Longgang Maternity and Child Institute of Shantou University Medical College (Longgang District Maternity & Child Healthcare Hospital of Shenzhen City), Clinical Laboratory, Shenzhen, China; 2 Guangzhou Women and Children’s Medical Center, Guangzhou Medical University, Clinical Laboratory, Guangzhou, Guangdong, China; 3 Charité – Universitätsmedizin Berlin, corporate member of Freie Universität Berlin and Humboldt – Universität zu Berlin, Institute of Microbiology, Infectious Diseases and Immunology, Berlin, Germany; Foundation for Innovative New Diagnostics, Geneve, Switzerland

**Keywords:** carbapenem-resistant enterobacteriaceae, antimicrobial drug resistance, enterobacteriaceae, children, multilocus sequence typing

## Abstract

Carbapenem-resistant Enterobacteriaceae (CRE) are a substantial health burden. Existing management guidelines are primarily based on clonal evidence. Therefore, epidemiological data are required. We evaluated the risk factors for CRE colonization and described the molecular and clinical characteristics of CRE colonization in pediatric inpatients in Guangzhou, China. Pediatric inpatients from three hospitals in Guangzhou, China, between July 2019 and January 2021 were included. Fecal samples were collected and screened for CRE. Microbiological cultures were grown on MacConkey agar plates, and bacteria were identified with a mass spectrometry microbial identification system. Antimicrobial susceptibility was tested with a VITEK2 system, and carbapenemase genes were amplified. A case-control study was conducted to evaluate the risk factors for fecal CRE colonization in pediatric inpatients. Of 4,033 patients screened, 158 (3.92%) were positive for CRE, including *Escherichia coli* (51.27%), *Klebsiella pneumoniae* (37.97%), and *Enterobacter cloacae* (6.96%). The most common carbapenemase genes were blaNDM-5 (51.89%), blaNDM-1 (15.19%), and blaIMP-4 (7.60%) and blaKPC-2 (3.80%). Hematological malignancies, respiratory diseases, otolaryngological diseases, nervous system diseases, oral administration of third-generation cephalosporins, and the combined use of two or more antibiotics were independently associated with CRE colonization. The most prevalent carbapenem-resistant *K. pneumoniae* sequence types (STs) were ST11 (8.06%), ST37 (8.06%), and ST76 (8.06%), and the most prevalent carbapenem-resistant *E. coli* STs were ST10 (9.88%), ST48 (13.58%), and ST58 (8.64%). Pediatric inpatients were colonized by a diversity of CRE strains. Infection control and prevention measures should be implemented to reduce CRE colonization.

**IMPORTANCE:**

This study assessed the clinical and molecular epidemiology of carbapenem-resistant Enterobacteriaceae in pediatric inpatients at three hospitals in South China by means of screening stool samples for carbapenem-resistant genes and a nested case-control study to determine risk factors for carriage of carbapenem-resistant Enterobacteriaceae. Of 4,033 fecal samples screened, 158 (3.92%) were positive for CRE, including *Escherichia coli* (51.27 %), *Klebsiella pneumoniae* (37.97%), and *Enterobacter cloacae* (6.96%). The most common carbapenemase genes harbored by gastrointestinal CRE strains were blaNDM-5, blaNDM-1, and blaIMP-4. Hematological malignancies, respiratory diseases, otolaryngological diseases, nervous system diseases, oral administration of third-generation cephalosporins, and the combined use of two or more antibiotics were independently associated with CRE colonization.

## INTRODUCTION

Carbapenems are considered effective treatments for multidrug-resistant Gram-negative bacterial infections (1). However, carbapenem-resistant (CR) Enterobacteriaceae (CRE) have emerged due to the widespread use of carbapenems over the past few decades and represent a threat to public health (2). Since the first identification of CR *Klebsiella pneumoniae* (CRKP) in 1996 in the United States, CRE has rapidly spread worldwide (3 – 5). In China, *Klebsiella pneumoniae* resistance to meropenem and imipenem grew from 2.9% and 3.0% in 2005 to 26.3% and 25% in 2018, an increase of more than the eightfold (6, 7). In Europe, CRKP is prevalent in the Balkan and Mediterranean countries with a prevalence of 30% in Romania, 40% in Italy, and 60% in Greece in 2014 (8). In addition, CRE infections are difficult to treat and are associated with higher mortality rates in vulnerable population (9). Therefore, surveillance and prevention of CRE are required.

There are three major resistance mechanisms in CRE: production of carbapenemases capable of hydrolyzing carbapenems, modification of the function of porins and other membrane-associated proteins, and activation of drug efflux pumps (10, 11). Carbapenemases found in CRE include Ambler class A β-lactamases such as *blaKPC* and *blaSIM*; Ambler class B metallo-β-lactamases, such as *blaNDM*, *blaIMP*, and *blaVIM*; and Ambler class D oxacillinases (OXAs), such as *blaOXA-232* and *blaOXA-48* (12). A previous study showed that the most prevalent genotypes in clinical isolates from China were *blaKPC* in *K. pneumoniae* and *blaNDM* in *Escherichia coli* (13).

CRE disseminates through the gastrointestinal tract and plays a crucial role in the transmission of antibiotic resistance (14). Gastrointestinal CRE colonization can result in life-threatening bloodstream infections, especially children with immunodeficiency or hematological diseases (15). Existing CRE management guidelines are generally based on clonal evidence, although the epidemiology of CRE colonization and clinical risk factors for CRE colonization in pediatric inpatients are not well characterized in South China. Therefore, we aimed to investigate the molecular and clinical epidemiology, evaluate the risk factors for the acquisition of gastrointestinal CRE colonization, and detect carbapenemases, extended-spectrum β-lactamases (ESBLs), and AmpC cephalosporinase (AmpC) in CRE strains isolated from fecal samples of pediatric inpatients.

## MATERIALS AND METHODS

### Sample collection and microbiological culture

We collected unique fecal samples from 4,033 inpatients aged ≤18 years between July 2019 and January 2021 from three hospitals in Guangzhou, China (Yuexiu District, Tianhe District, and Zengcheng District), as previously described (16). The fecal samples were individually streaked onto selective MacConkey agar plates with 1.5 µg/mL meropenem added (Macklin, China). After 48 h of incubation at 5% CO_2_ at 35°C, suspected CRE colonies from each MacConkey agar plate were isolated and streaked on blood agar plates for further confirmation. All positive strains were maintained at −80°C for further investigation.

### Microbial identification and antibiotic susceptibility testing

All colonies that grew on the blood agar plates were analyzed using an automated mass spectrometry microbial identification system (VITEK MS, bioMérieux, Marcy-l’Étoile, France). Susceptibility to 16 antimicrobial agents (cefoperazone + sulbactam, piperacillin + tazobactam, meropenem, imipenem, ceftazidime, cefepime, aztreonam, amikacin, tobramycin, ciprofloxacin, levofloxacin, trimethoprim + sulfamethoxazole, doxycycline, tetracycline, minocycline, and colistin) was assessed using a VITEK2 Compact System (bioMérieux) with VITEK 2 AST-N335 cards. Antibiotic susceptibility testing was performed, and the results were interpreted according to the most recent Clinical and Laboratory Standards Institute (CLSI) Guidelines at the time (CLSI 2019 to CLSI 2021). *Escherichia coli* (ATCC25922) and *Klebsiella pneumoniae* (ATCC700603) strains were used for quality control.

### Clinical data collection

We conducted a case-control study to identify risk factors for gastrointestinal CRE colonization in pediatric inpatients. Among the 4,033 patients investigated, review of medical record was completed for 3,486 patients, including all 158 CRE carriers and 3,328 non-CRE carriers. We were unable to obtain the medical records of 547 non-CRE carriers and, therefore, excluded these patients from the case-control study. Data on general characteristics, length of hospitalization, and previous use of antibiotics (particularly carbapenems, β-lactam + β-lactamase inhibitors, and extended-spectrum cephalosporins) were collected from the electronic patient management system for all 3,486 patients. The non-CRE carriers were used as the control group.

### DNA extraction and detection of antimicrobial resistance genes

A commercially available DNA extraction kit (Steady Pure Bacterial Genomic DNA Extraction Kit, Accurate Biotechnology Co. Ltd., China) was used to extract DNA according to the manufacturer’s instructions. We used polymerase chain reaction (PCR) and Sanger sequencing to screen for carbapenemase genes (*blaKPC*, *blaNDM*, *blaIMP*, *blaGES*, *blaVIM*, *blaSIM*, *blaOXA-23*, *blaOXA-48*, *blaGIM*, and *blaSPM*) (17), ESBL (*blaTEM*, *blaSHV*, and *blaCTX-M*), Amp C β-lactamases (*blaMOX*, *blaDHA*, *blaCMY*, *blaACC*, *blaCIT*, and *blaEBC*), and *mcr-1* (18, 19). The modified carbapenem inactivation method (m-CIM manufacturer) (20) was used to confirm carbapenemase production.

### Molecular typing

Multilocus sequence typing (MLST) was performed on *K. pneumoniae* and *E. coli* isolates according to the methods described on the Pasteur Institute MLST website (https://bigsdb.pasteur.fr/cgi-bin/bigsdb/bigsdb.pl?db=pubmlst_klebsiella_seqdef) and the Pub MLST website (https://pubmlst.org/). Housekeeping genes of *K. pneumoniae* (*ropB*, *gapA*, *phoE*, *mdh*, *pgi*, *infB*, and *tonB*) and *E. coli* (*adk*, *mdh*, *fumC*, *gyrB*, *icd*, *purA*, and *recA*) were amplified and sequenced (21, 22). The sequence types (STs) were determined by comparing the sequences with those in the MLST database. A minimum spanning tree of 60 CRKP and 81 CR *E. coli* strains were constructed using BioNumerics software version 7.6 (bioMérieux).

### Statistical analysis

SPSS software 26.0 (IBM Corp., Armonk, NY, USA) was used for statistical analysis. Chi-square tests were performed to identify risk factors for CRE colonization. Factors showing *P* < 0.05 were considered as candidate predictors that were significantly related to CRE isolation Multivariable logistic regression was performed including these factors in the model to estimate odds ratios (ORs) with 95% confidence intervals (CIs).

## RESULTS

### CRE in fecal samples

CRE was isolated from 158 (3.92%) of the 4,033 fecal samples screened, comprising *Escherichia coli* (*n* = 81, 51.27%), *Klebsiella pneumoniae* (*n* = 60, 37.97%), *Enterobacter cloacae* (*n* = 11, 6.96%), *Citrobacter freundii* (*n* = 4, 2.53%), *Klebsiella aerogenes* (*n* = 1, 0.63%), and *Klebsiella oxytoca* (*n* = 1, 0.63%) (Table 1).

**TABLE 1:** Number of CRE strains isolated from fecal samples of pediatric inpatients

Isolate species	*n*	%
*Escherichia coli*	81	51.27
*Klebsiella pneumoniae*	60	37.97
*Enterobacter cloacae*	11	6.96
*Citrobacter freundii*	4	2.53
*Klebsiella aerogenes*	1	0.63
*Klebsiella oxytoca*	1	0.63
Total	158	100

### Antibiotic susceptibility

All CRE isolates were susceptible to tigecycline and colistin (100%) (Table 2). All CRE isolates were highly resistant to the third- or fourth-generation cephalosporin and β-lactam + β-lactamase inhibitor (>95%). The prevalence of resistance to aztreonam, amikacin, tobramycin, levofloxacin, and ciprofloxacin was 44.94%, 12.25%, 22.78%, 53.80%, and 64.56%, respectively.

**TABLE 2:** Antibiotic susceptibility of CRE isolates from fecal samples of pediatric inpatients

	CRE (*n* = 158)		*E. coli* (*n* = 81)		*Klebsiella pneumoniae* (*n* = 60)	
**Antimicrobial agents**	**R,^*a*^ *n* (%)**	**S,^*b*^ *n* (%)**	**R, ^*a*^ *n* (%)**	**S, ^*b*^ *n* (%)**	**R, ^*a*^ *n* (%)**	**S, ^*b*^ *n* (%)**
Piperacillin/tazobactam	157 (99.37)	1 (0.93)	80 (100)	0 (0)	61 (100)	0 (0)
Cefoperazone/sulbactam	157 (99.37)	1 (0.93)	80 (100)	0 (0)	61 (100)	0 (0)
Ceftazidime	157 (99.37)	1 (0.93)	80 (100)	0 (0)	61 (100)	0 (0)
Cefepime	154 (97.47)	2 (1.27)	80 (100)	0 (0)	58 (96.88)	1 (1.04)
Aztreonam	71 (44.94)	87 (55.06)	29 (36.25)	51 (63.75)	34 (55.74)	27 (44.26)
Imipenem	154 (97.47)	1 (0.93)	80 (99.29)	0 (0)	57 (93.44)	1 (1.64)
Meropenem	158 (100)	0 (0.00)	80 (100)	0 (0)	61 (100)	0 (0)
Amikacin	19 (12.25)	137 (86.71)	10 (8.00)	69 (86.25)	10 (6.10)	50 (81.97)
Tobramycin	36 (22.78)	77 (48.73)	25 (31.25)	29 (36.25)	19 (18.33)	35 (57.38)
Levofloxacin	85 (53.80)	36 (22.78)	54 (67.50)	11 (13.75)	28 (45.90)	19 (31.15)
Ciprofloxacin	102 (64.56)	47 (31.01)	60 (75.00)	17 (21.25)	36 (59.02)	21 (34.43)
Trimethoprim/sulfamethoxazole	113 (71.51)	45 (28.48)	68 (85.00)	12 (17.02)	33 (54.10)	28 (45.90)
Minocycline	85 (53.80)	30 (18.99)	52 (65.00)	14 (17.50)	28 (45.90)	14 (22.95)
Tigecycline	0 (0)	158 (100）	0 (0)	80 (100)	0 (0)	61 (100)
Doxycycline	107 (67.72)	36 (22.78)	65 (81.25)	8 (10.00)	34 (55.74)	21 (34.43)
Colistin	0 (0)	158 (100）	0 (0)	80 (100)	0 (0)	61 (100)

^*a*^ R, resistant.

^*b*^ S, susceptible.

### Clinical characteristics and risk factors

Table 3 shows the clinical characteristics and risk factors of CRE colonization. The median age of children with gastrointestinal CRE colonization and control subjects was 23.5 and 14.0 months, respectively. The median length of hospitalization was 21 and 7 days, respectively . The detection rate of CRE varied by department: hematology (17.48%, 25/168), respiratory (8.28%, 14/169), intensive care unit (6.07%, 33/544), gastroenterology (3.35%, 8/239), and cardiovascular (1.61%, 3/186) (Table 3). Logistic regression analysis revealed that several variables were associated with CRE colonization. Compared to the control group, patients with CRE colonization were more likely to have hematological malignancies (OR, 2.233; 95% CI, 1.088–4.540; *P* = 0.028), respiratory diseases (OR, 4.129; 95% CI, 2.545–6.697; *P* < 0.001), otolaryngological diseases (OR, 2.195; 95% CI, 1.073–4.491; *P* = 0.031), and nervous system diseases (OR, 8.727; 95% CI, 4.462–17.304; *P* < 0.001). Moreover, surgery (OR, 10.599; 95% CI, 6.660–16.868; *P* < 0.001), intubation/mechanical ventilation (OR, 2.391; 95% CI, 1.341–4.266; *P* = 0.003), bone marrow biopsy (OR, 6.791; 95% CI, 3.019–15.275; *P* < 0.001), oral administration of third-generation cephalosporins (OR, 2.053; 95% CI, 1.290–3.268; *P* < 0.001), and combined use of two or more antibiotics (OR, 7.763; 95% CI, 5.095–11.827; *P* < 0.001) were significantly associated with CRE colonization.

**TABLE 3:** Clinical characteristics of CRE carriers

Characteristics	CRE (*n* = 158) (N/median) (%/IQR)	Control (non-CRE carriers) (*n* = 3328) (N/median) (%/IQR)	Univariate analysis			
			OR (95% CI)	*P*-value	OR (95% CI)	*P*-value
Age (mo）	23.50 (12–62.50）	14 (1–51）	0.896 (0.590–1.360)	0.606		
Male gender	100 (4.82)	1,974 (95.18)	1.001 (0.996–1.006)	0.715		
Length of stay (d)	21 (6–29)	7 (5–12)	0.992 (0.980–1.004)	0.174		
Department						
ICU	33 (6.06)	511 (93.93)	2.194 (0.905–5.270)	0.876		
PICU	11 (7.48)	136 (92.51)	1.843 (0.118–28.880)	0.663		
CICU	6 (7.59)	73 (92.40)	0.221 (0.032–2.221)	0.221		
NICU	16 (5.03)	302 (94.96)	2.989 (0.191–46.786)	0.435		
Cardiovascular	3 (1.61)	183 (98.39)	0.620 (0.224–0.224-1.715)	0.357		
Hematology	25 (14.88)	143 (85.12)	0.235 (0.058–0.944)	0.041		
Respiratory	14 (8.28)	155 (91.71)	0.581 (1.249–2.752)	0.581		
Gastroenterology	8 (3.35)	231 (96.65)	1.825 (0.611–5.450)	0.281		
Neonatal pediatrics	6 (1.10)	539 (98.90)				
Underlying condition						
Gastrointestinal diseases	34 (5.80)	552 (94.20)	0.971 (0.484–1.948)	0.933		
Hematological malignancies	30 (19.0)	221 (81.00)	5.482 (1.585–18.958)	0.007	2.233 (1.088–4.540)	0.028
Cardiovascular disease	9 (3.21)	271 (96.79)	5.482 (1.585–18.958)	0.755		
Respiratory disease	59 (8.99)	597 (91.01)	7.762 (4.198–14.350)	<0.001	4.129 (2.545–6.697)	<0.001
Nervous system disease	19 (13.48)	122 (86.52)	11.052 (4.393–27.809)	<0.001	8.727 (4.462–17.304)	<0.001
Liver disease	5 (3.42)	141 (96.57)	0.973 (0.362–2.616)	0.956		
Otolaryngological disease	6 (4.58)	125 (95.42)	2.767 (1.193–6.419)	0.018	2.195 (1.073–4.491)	0.031
Invasive procedures						
Surgery	65 (13.43)	419 (86.57)	9.610 (5.488–16.827)	<0.001	10.599 (6.660–16.868)	<0.001
Intubation/mechanical ventilate	26 (16.04)	136 (83.96)	2.213 (1.120–4.375)	0.022	2.391 (1.341–4.266)	0.003
Bone marrow puncture	27 (36.99)	46 (63.01)	12.084 (4.590–31.810)	<0.001	6.791 (3.019–15.275)	<0.001
Central venous catheter	10 (7.30)	127 (92.70)	0.699 (0.211–2.317)	0.558		
Lumbar puncture	5 (4.10)	117 (95.90)	0.561 (0.201–1.563)	0.269		
Abdominal drainage	37 (20.67)	142 (79.39)	2.077 (0.908–4.751)	0.083		
Antibiotic exposures						
β-Lactam + β-lactamase inhibitor	80 (16.77)	397 (83.23)	1.345 (0.747–2.420)	0.323		
First- and second-generation cephalosporins	38 (10.27)	332 (89.73)	0.955 (0.548–1.662)	0.87		
Third-generation cephalosporins	66 (18.23)	296 (81.77)	6.495 (3.334–12.650)	<0.001	2.053 (1.290–3.268)	<0.001
Carbapenems	58 (23.20)	192 (76.80)	1.649 (0.911–2.983)	0.098		
Macrolides	20 (3.06)	634 (96.94)	1.345 (0.915–2.917)	0.094		
Glycopeptide	34 (24.11)	107 (75.89)	0.879 (0.457–1.693)	0.7		
Combined use of two or more antibiotics	83 (24.27)	259 (75.73)	3.421 (1.875–6.242)	<0.001	7.763 (5.095–11.827)	<0.001
Outcome						
Cure	153 (4.42)	3,309 (95.58)				
Death	5 (20.83)	19 (79.17)				

### Antimicrobial resistance genes

Among the 158 CRE-colonized patients, 145 were tested positive using the mCIM assay. Carbapenemase genes were detected in 134 of 158 CRE isolates using PCR and Sanger sequencing. Carbapenemase genotypes were not detected in seven *E. coli*, two *E. cloacae*, and one *K. pneumoniae* isolates. *blaNDM-5* (51.89%, 82/158) and *blaNDM-1* (15.19%, 24/158) were predominant carbapenemase genes among all strains, followed by *blaIMP-4* (7.60%, 12/158) and *blaKPC-2* (3.80%, 6/158) (Table 4). Thirteen CRE isolates harbored two different carbapenemase genes, including one *K. pneumoniae* harboring *blaKPC-2* and *blaNDM-5*, two *E. coli*, and seven *K. pneumoniae* harboring *blaIMP-4* and *blaNDM-1*. There is one *blaGES-1 E. coli* detected in our study. We did not detect the *blaSIM*, *blaOXA-23*, *blaOXA-48*, or *mcr-1* genes. ESBL genes, such as *blaCTX-M* (44.94%, 71/158), *blaTEM* (84.81%, 134/158), *blaSHV* (33.54%, 53/158), and *AmpC* genes, including *blaDHA* (12.66%, 20/158) and *blaFOX* (25.32%, 40/158), were also detected in these CRE isolates.

**TABLE 4:** Prevalence of carbapenemase genes among 158 CRE strains isolated from fecal samples of pediatric inpatients

Isolates	*E. coli*	*K. pneumoniae*	*E. cloacae*	*C. freundii*	*K. aerogenes*	*K. oxytoca*	Total
	80 (50.64)	61 (38.61)	11 (6.96)	4 (2.53)	1 (0.63)	1 (0.63)	158
Carbapenemase phenotypic confirmatory test positive (*n*)	79	51	10	3	1	1	145
KPC–2	–	3	–	–	–	–	3
NDM–1	16	7	–	–	–	1	24
NDM–5	51	21	6	3	1	–	82
NDM–5 + KPC–2	–	1	–	–	–	–	1
NDM–1 + IMP–4	2	7	–	–	–	–	9
IMP–4	4	17	3	4	–	–	13
VIM–1	–	–	1	–	–	–	1
OXA23	–	1	–	–	–	–	1
OXA48	–	–	–	–	–	–	0
GES–1	1	–	–	–	–	–	1
SIM	–	–	–	–	–	–	0
ther	7	1	2	–	–	–	10
mcr–1	–	–	–	–	–	–	0

### Characteristics of sequence types

**Fig 1:**
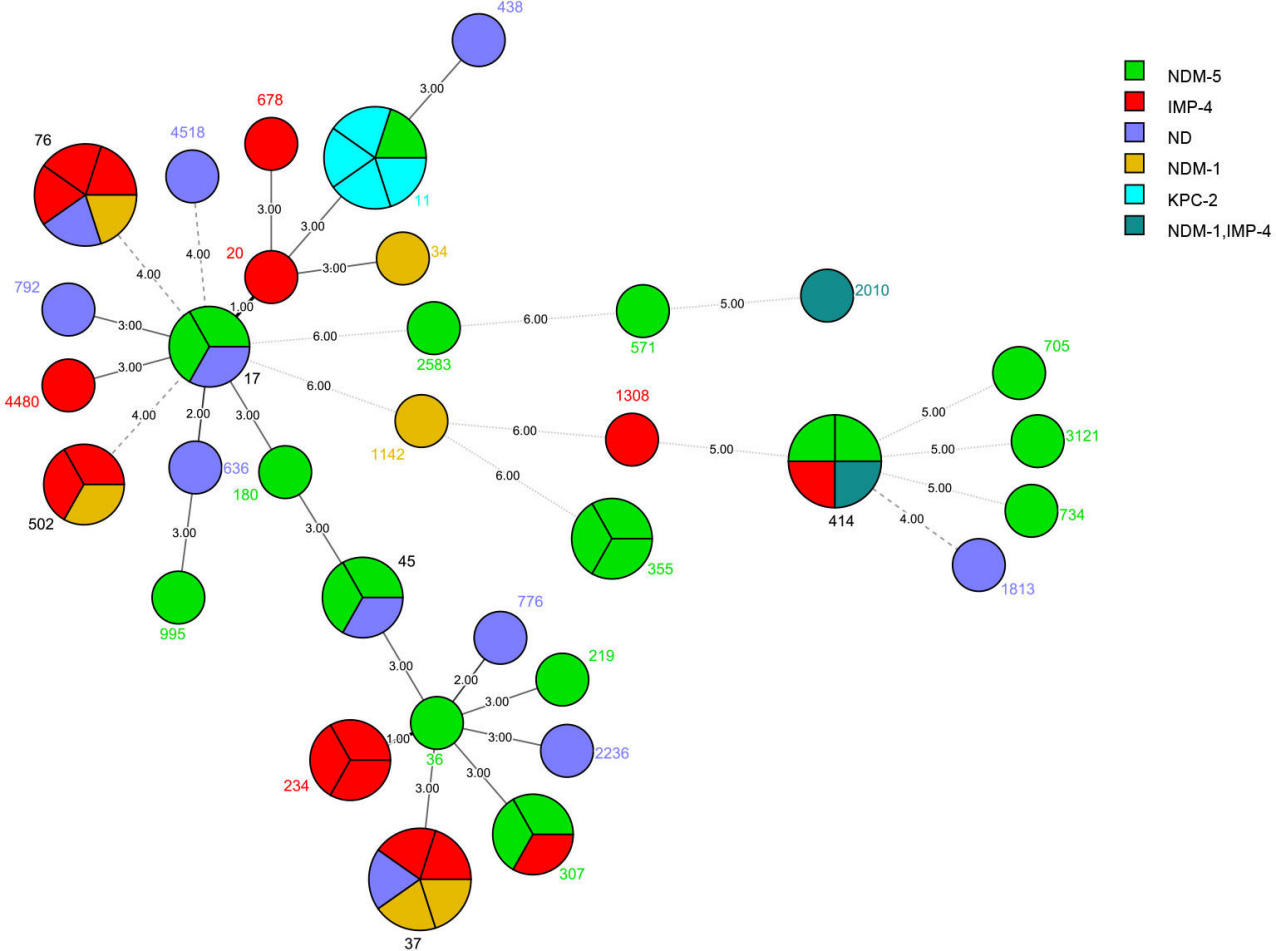
Phylogenetic tree of multilocus sequence typing data from carbapenem-resistant *Klebsiella pneumoniae* isolates.

**Fig 2:**
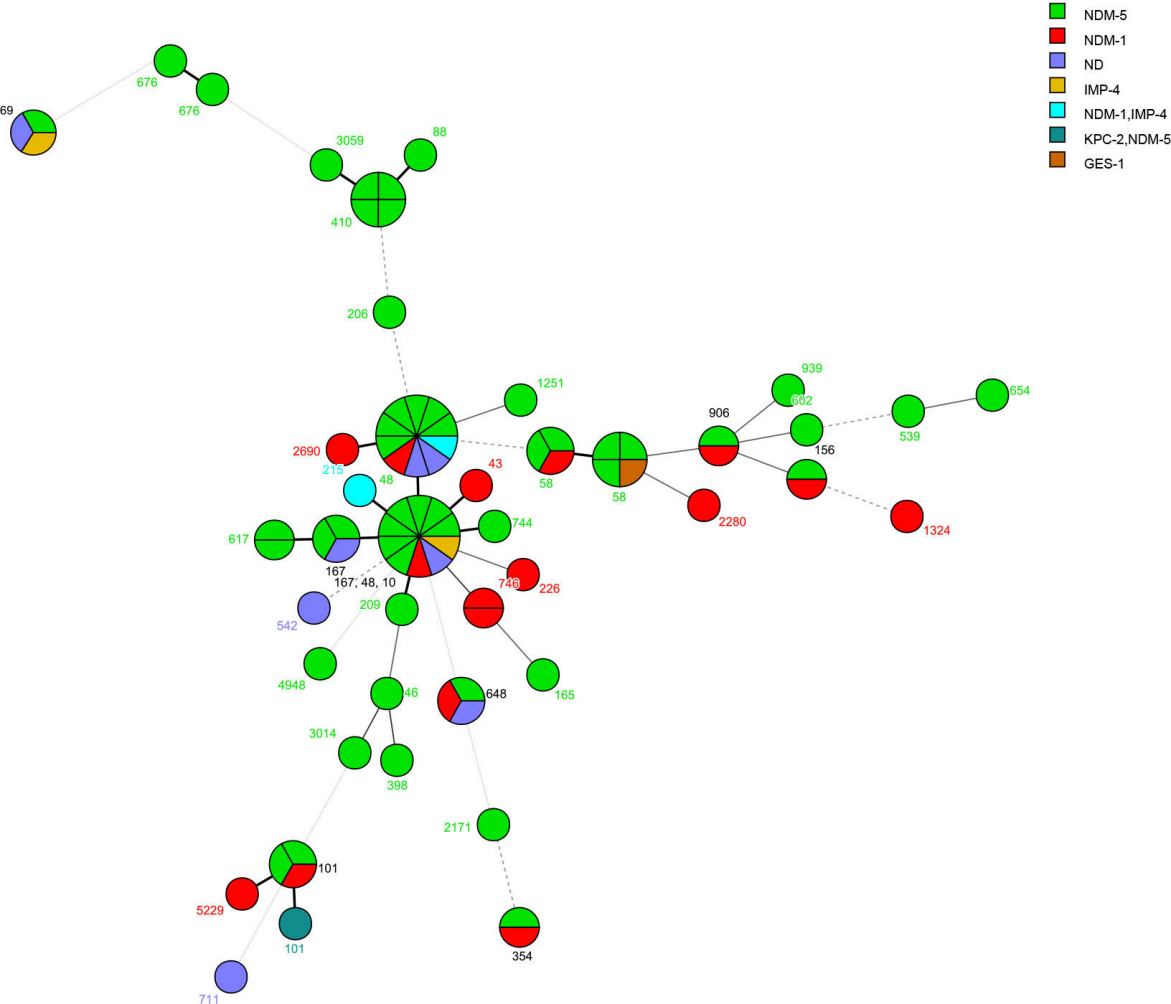
Phylogenetic tree of multilocus sequence typing data from carbapenem-resistant *Escherichia coli* isolates.

A total of 33 different STs were identified among the 62 CRKP strains (Fig. 1 and 2). The four most prevalent STs among CRKP isolates were ST11 (8.06%), ST37 (8.06%), ST76 (8.06%), and ST414 (6.45%), comprising 30.63% of all CRKP strains. Thirty-nine different STs were identified in the 81 CR *E. coli* strains. The three most prevalent STs among *E. coli* were ST10 (9.88%), ST48 (13.58%), and ST58 (8.64%), comprising 32.10% of all CR *E. coli*. Uncommon STs in *K. pneumoniae* and *E. coli* were identified in one or two strains each, as shown in Fig. 1 and 2.

Figures 1 and 2 also illustrate the carbapenemase distribution among the different STs. In *K. pneumoniae*, *blaKPC-2* was detected in ST11, whereas *blaNDM-5* was detected in ST11, ST17, ST89, ST45, ST180, ST219, ST307, ST355, ST414, ST571, ST705, ST734, ST995, ST2583, and ST3121. In addition, the isolates carrying both *blaNDM-1* and *blaIMP-4* were classified as ST414 and ST2010, and the isolates carrying *blaOXA-23* were classified as ST1813. In *E. coli*, the dominant carbapenemase was *blaNDM-5*, which were found in isolates belonging to more varied ST types than other carbapenemase gene (Fig. 2). *blaNDM-1* was detected in ST43, ST48, ST58, ST101, ST156, ST226, ST354, ST648, ST746, ST906, ST1324, ST2280, ST2690, and ST5229. Isolates carrying both *blaNDM-1* and *blaIMP-4* were classified as ST48 and ST215, whereas isolates carrying *blaKPC-2* and *blaNDM-5* were classified as ST101. *blaGES-1* was detected in ST58, and *blaIMP-4* was detected in ST69 and ST10.

## DISCUSSION

In this multicenter analysis of CRE in pediatric inpatients hospitalized in Guangzhou, we determined the molecular epidemiology and identified risk factors for CRE colonization. The carriage rate of CRE among pediatric patients varies in different geographic areas (23). In this study, the fecal CRE carriage rate in pediatric inpatients was 3.92%, which is lower than that of three other hospitals in Hunan (8.5%), Fujian (6.6%), and Shanghai (8.6%) (23 – 25). There are several possible reasons that could contribute to this phenomenon. First, inpatients from neonatology (15.63%, 545/3486) comprised a large part of the inpatient population in our study. The fecal CRE colonization rate in the newborn population is usually lower, due to less exposure to antibiotics. Second, seasonal and temperature variations can affect CRE detection in fecal samples (26). Although the CRE colonization rate is dynamic owing to seasonal variations, geographical region, and health state, further research is needed to better identify the causes of these disparities.

Globally, as in China, carbapenemase production is the primary mechanism used by Enterobacteriaceae to resist carbapenems (27). Our research confirmed this, as 145 (91.77%) of our CRE isolates were positive in the *mCIM assay*. In a 5-year study on CRKP conducted in a pediatric hospital in China, *blaIMP-4* was the most common carbapenemase from 2010 to 2012, whereas *blaNDM-1* and *blaNDM-5* were the most common carbapenemases in 2018 (7, 28), which is consistent with our findings. Notably, *blaKPC-2* gene was only detected in four isolates: three CRKP strains and one CR *E. coli* strain. *blaKPC-2* is the most prevalent carbapenemase in the adult population (29). These results suggest that anti-CRE treatment strategies for children should differ from those used in adults. In addition, we found that two *K. pneumoniae* strains and one *E. coli* strain carried *blaIMP-4* and *blaNDM-5*, and one *E. coli* strain carried *blaNDM-5* and *blaKPC-2* simultaneously, suggesting that these carbapenemase genes are readily acquired.

All CRE isolates in our study were multidrug resistant and showed high resistance to carbapenems and cephalosporins. The prevalence of resistance to amikacin, tobramycin, colistin, and tigecycline was low, which may be because of limited use of these antibiotics in children. This suggests that tigecycline or colistin, in conjunction with other antibiotics such as meropenem and imipenem, may be useful for the treatment of complex CRE infections. In recent years, avibactam-ceftazidime, a novel cephalosporin/β-lactamase inhibitor combination, has been used to inhibit the activities of carbapenemase (30) but it is less effective at inhibiting in Ambler class B carbapenemases such as *blaNDM* (NDM) (31). The use of avibactam-ceftazidime in our region’s medical center for children should be carefully considered.

The case-control analysis revealed that several serious diseases are associated with CRE colonization, including hematological malignancies, respiratory disease, otolaryngological disease, and nervous system disease. Among patients with hematological malignancies, respiratory disease, and nervous system disease, CRE colonization was associated with immunosuppression and high use of antibiotics. It is noteworthy that otolaryngological diseases are a risk factor for CRE colonization at our medical center, as previous studies have not identified otolaryngological disease as a risk factor for CRE colonization. Therefore, it is important to understand the prevalence of CRE in the otolaryngology departments. Additionally, physicians should consider the possibility that a patient may have CRE if risk indicators are present. Our study also showed that oral administration of third-generation cephalosporins and the combined use of two or more antibiotics were risk factors for CRE colonization, which is consistent with the findings of other studies (32 – 34). Surprisingly, oral administration of carbapenems and β-lactam + β-lactamase inhibitor was not a risk factor for CRE colonization in this study. This may be due to the sample size of our study or missing information during extraction of data from the electronic medical system. Therefore, the influence of varied antibiotic exposure on gastrointestinal CRE colonization requires further study.

The MLST results showed a high level of genetic diversity among *K. pneumoniae* and *E. coli* isolates, with 60 *K*. *pneumoniae* strains divided into 32 STs and 81 *E. coli* strains divided into 39 STs. The most prevalent *K. pneumoniae* STs were ST11, ST37, and ST414. Four of the five ST11 *K. pneumoniae* isolates carried the *blaKPC-2* gene. *K. pneumoniae* ST11 carrying *blaKPC-2* has been identified as the major type in another area of China, which suggests that clonal transmission of ST11 CRKP occurred in the hospitals in this study (35). NDM-5-producing *K. pneumoniae* had different clonal backgrounds; *blaNDM-1* was observed in ST17, ST36, ST45, and ST180 isolates. The most prevalent *E. coli* STs in this study were ST48, ST10, and ST58, and *blaNDM-5* was more prevalent in the *E. coli* group. Notably, *E. coli* ST48 and ST101 co-harbored *blaNDM-1* and *blaIMP-4*, and a novel *K. pneumoniae* ST2010 co-harbored *blaNDM-1* and *blaIMP-4*.

This study has some limitations. First, stool samples may not be representative because the stool samples were restricted to those submitted for routine analysis. It would be worthwhile to assess the CRE situation in the study facilities. Second, because we used only one method for molecular CRE characterization some CRE strains may have been missed.

In conclusion, the CRE carriage rate in pediatric inpatients in South China was 3.92%, with a diversity of STs found in CRKP and CR *E. coli*. Moreover, *blaNDM-5* and *blaNDM-1* were the most prevalent resistance genes in CRE isolates. Hematological malignancies, respiratory disease, otolaryngological disease, nervous system disease, oral administration of third-generation cephalosporins, and combined use of two or more antibiotics were independently associated with CRE colonization. Physicians should consider the possibility of a patient carrying CRE if risk indicators are present. This study provides insight into the current status of fecal carriage of CRE among pediatric inpatients in South China. Implementation of prevention and control measures in clinical settings should be encouraged based on our findings.

## Data Availability

The data sets generated for this study can be found in the Institut Pasteur (https://bigsdb.pasteur.fr/cgi-bin/bigsdb/bigsdb.pl?db=pubmlst_klebsiella_seqdef&page=downloadAlleles&tree=1) and the Pub MLST website (https://pubmlst.org/bigsdb?db=pubmlst_escherichia_seqdef&page=downloadAlleles&tree=1).
